# Do Patients on Section 42 Understand Their Section and What Is Involved?

**DOI:** 10.1192/bjo.2022.342

**Published:** 2022-06-20

**Authors:** Chung Mun Alice Lin, Neeti Sud

**Affiliations:** 1 National Institute of Health Research Newcastle Biomedical Research Centre and the Translational and Clinical Research Institute, Newcastle University, Newcastle upon Tyne, United Kingdom; 2 Cumbria, Northumberland, Tyne and Wear NHS Foundation Trust, Newcastle upon Tyne, United Kingdom

## Abstract

**Aims:**

Mental health disorders, mostly notably paranoid schizophrenia and personality disorders are commonly seen in patients with a forensic background. Section 37/41, within the Mental Health Act 1983, detains patients who are mentally unwell in hospital for treatment, instead of a prison sentence, with the addition of a community restriction order for public safety. Once stable, patients are discharged by the Ministry of Justice (MoJ) on Section 42, otherwise known as a conditional discharge. This means they can live freely in the community but under a set of conditions they must follow in order to obtain absolute discharge. A leaflet on Section 42 was created after a gap in patient knowledge was identified during consultations. Furthermore, a literature review did not retrieve any relevant results on this topic. The aim of this leaflet was to improve both patient and staff knowledge.

**Methods:**

A patient leaflet was created using information from relevant legislation, MoJ official documents, trust resources, the charity MIND UK as well as staff knowledge. A checklist consisting of 12 questions was created to test the patients’ knowledge, with space for additional comments. Care was taken to ensure every question on the checklist had a corresponding answer in the leaflet. Six suitable patients were identified and supported to read the leaflet and a structured interview using the checklist was conducted pre- and post-leaflet. In addition, feedback was sought from staff members of multiple backgrounds. A resource questionnaire was also given to participants to collate feedback. The pre- and post-test answers were compared and given a mark out of 12. A mark was given for answers that were sensible and correct, even if parts were missing for questions that encompassed multiple facts.

**Results:**

All patients were previously on Section 37/41 and now on Section 42. All showed a substantial improvement in knowledge base, with 4/6 patients scoring full marks afterwards. Patient feedback obtained was overall very positive, with many describing it as “useful”, “informative” and “helpful”. Staff feedback was also collated and found to be positive too, with comments including “very informative”, “easy to read” and “clear and precise”.

**Conclusion:**

Our leaflet was well received by both patients and staff. It improved their knowledge base as well as confidence in understanding the medico-legal jargon used in day-to-day practice in the forensic setting. Feedback was overall positive, and the additional patient feedback was encouraging, with many of them wishing for sooner access to similar resources.

